# Screening and verification of genes associated with leaf angle and leaf orientation value in inbred maize lines

**DOI:** 10.1371/journal.pone.0208386

**Published:** 2018-12-07

**Authors:** Shi Lu, Mo Zhang, Zhuo Zhang, Zhenhui Wang, Nan Wu, Yang Song, Piwu Wang

**Affiliations:** Jilin Agricultural University, Chang Chun, China; Universidad Miguel Hernández de Elche, SPAIN

## Abstract

Leaf angle and leaf orientation value are important traits affecting planting density and photosynthetic efficiency. To identify the genes involved in controlling leaf angle and leaf orientation value, we utilized 1.49×10^6^ single nucleotide polymorphism (SNP) markers obtained after sequencing 80 backbone inbred maize lines in Jilin Province, based on phenotype data from two years, and analyzed these two traits in a genome-wide association study (GWAS). A total of 33 SNPs were significantly associated (P<0.000001) with the two target traits. Twenty-two SNPs were significantly associated with leaf angle and distributed on chromosomes 1, 3, 4, 5, 6, 7, 8, and 9, explaining 21.62% of the phenotypic variation. Eleven SNPs were significantly associated with leaf orientation value and distributed on chromosomes 1, 3, 4, 5, 6, 7, and 9, explaining 29.63% of the phenotypic variation. Within the mean linkage disequilibrium (LD) distance of 9.7 kb for the significant SNP locus, 22 leaf angle candidate genes were detected, and 3 of these candidate genes harbored significant SNPs, with phenotype contribution rates greater than 10%. Two candidate genes at distances less than 100 bp from significant SNPs showed phenotype contribution rates greater than 8%. Seven leaf orientation value candidate genes were detected: 3 of these candidate genes harbored significant SNPs, with phenotype contribution rates greater than 10%. Eight inbred maize lines with significant differences in leaf angle and leaf orientation value were selected to test candidate gene expression levels from 182 recombinant inbred lines (RILs). The 5 leaf angle candidate genes and 3 leaf orientation value candidate genes were verified using quantitative real-time PCR (qRT-PCR). The results showed significant differences in the expression levels of the above eight genes between inbred maize lines with significant differences in leaf angle and leaf orientation value.

## Introduction

The characteristics of maize plant types, including the leaf angle, leaf orientation value, leaf length, and leaf width, are associated with the spatial distribution of maize [1,2]. The more upright the upper leaves of the ear are, the stronger the density tolerance of the plant. Erect leaves effectively contribute to the maize grain yield by enhancing the photosynthetic utilization rate, which is an important factor in increasing population yield in maize [3,4]. The genetic study of maize plant types is an important basis for density-tolerant maize breeding [5], and in recent decades, plant type has been the focus of maize breeding [6]. The use of parental mapping to locate maize plant type quantitative trait loci (QTLs) has previously been reported. Mickelson et al. [7] identified 9 leaf angle QTLs that were distributed on chromosomes 1, 2, 4, 5, 6 and 7 in two environments using an RFLP technique in the B73 × Mo17 population with 180 recombinant inbred lines (RILs). Pelleschi et al. [8] identified 2 leaf length QTLs and 3 leaf width QTLs using an RFLP technique to analyze the F-2 × MBS847 population. Using 194 pairs of SSR primers, Zhang et al. [9] identified 33 plant height QTLs and 37 ear height QTLs among two populations: Qi 319 × Huangzaosi (Q/H) and Ye 478 × Huangzaosi (Y/H). However, reflecting the limitations of low density and a large confidence interval, these published QTLs were primarily confined to the positioning level, and it is difficult to further screen and verify candidate genes.

Genome-wide association studies (GWASs) use millions of SNPs in the genome as molecular genetic markers for comparative and correlation analyses at the genome-wide level [10,11]. GWASs have the advantages of high-resolution and high-throughput analysis and are suitable for the identification of favorable alleles in germplasm resources [12,13]. GWASs have been applied to genetic studies of quantitative characters in rice, *Arabidopsis*, maize and other crops [14]. Huang et al. [15] detected 950 rice varieties in a GWAS, revealing 32 SNPs significantly associated with flowering and 10 SNPs significantly associated with yield. Aranzana et al. [16] also conducted a GWAS and identified a new type of defense gene in *Arabidopsis* under propylene stress. Using a population of 236 maize inbred lines and a Maize SNP50 Beadchip, Liu et al. [17] detected 73 SNPs significantly associated with maize rough dwarf disease resistance, and 41 candidate genes were identified in the linkage disequilibrium (LD) region. Tian et al. [18] and Buckler et al. [19] used a nested association mapping (NAM) population to conduct joint linkage mapping to examine maize leaf architecture, identifying 36 QTLs for leaf length, 30 QTLs for leaf angle, and 34 QTLs for leaf width. However, none of the above studies verified the candidate genes. In the present study, 80 backbone inbred maize lines in Jilin Province were used as experimental materials for next-generation sequencing (NGS) technology to screen for SNPs associated with leaf angle and leaf orientation value and to detect and verify related functional genes, thereby enriching the molecular genetic basis of plant type traits in maize.

## Materials and methods

### Construction of the study population

GWAS population: A total of 80 parental inbred lines obtained from a maize hybrid total planting area of more than 80% in Jilin Province were collected over the past 5 years as the GWAS population material and planted in the experimental field of Jilin Agricultural University in 2015 and 2016. The population had a random block design, with each plot comprising 3 lines of 3 m in length planted 0.6 m apart. The experiments were repeated 3 times.

Biparental mapping population: The leaf compact type inbred line P014 and the leaf explant inbred line E1312 were used as the parents, and hybridization and continuous inbreeding were performed for 7 generations. Biparental mapping population, comprising 180 recombined inbred lines that were used to verify candidate genes for the control of leaf angle and leaf orientation value, was planted in the experimental field of Jilin Agricultural University in 2015 and 2016. The population had a random block design, with each plot comprising 3 lines of 3 m in length planted 0.6 m apart. The experiments were repeated 3 times.

### Statistics and analysis of leaf angle and leaf orientation value phenotype data

During the formation stage of plant type (R1), the leaf angle and leaf orientation value were measured for each inbred line, and 5 plants were continuously selected between the borderlines. The data were analyzed using SPSS19.0 software and the following specific determination criteria.

Leaf angle (LA): Using a protractor, for the leaves of the upper ear, the acute angle between the blade and the stem was measured, and the average number was calculated.

Leaf orientation value (LOV): For the leaves of the upper ear, the leaf angle coangle was multiplied by the ratio of the length from the ligule to the flagging point of the measured leaf to the leaf length, and the average number was calculated.

The formula was suggested by Pepper [2]:

LOV=∑(90‑θi)×(Lf/L)/N

where θi is the leaf angle, Lf is the distance from the base of the ligule to the flagging point of the measured leaf, L is the leaf length, and N is the number of measured leaves.

### Whole-genome NGS

Genomic DNA was extracted from the GWAS population using a modified CTAB [20] method. The data were sent to the Novogene Biological Company (Beijing, China) for NGS. The DNA sample was fragmented by sonication to a size of 350 bp, and the DNA fragments were subjected to end polishing and A-tailing and subsequently ligated to the full-length adapter for Illumina sequencing. The libraries were analyzed in terms of size distribution using an Agilent2100 Bioanalyzer. Sequencing libraries were generated using the TruSeq Nano DNA HT Sample Preparation Kit (Illumina USA), and index codes were added to attribute sequences to each sample. The above-constructed libraries were sequenced using the Illumina HiSeq PE150 platform.

Original image data were transformed into raw reads by CASAVA base calling, and the result was saved in fastq format (*.fq). After removing the adapter and unrecognized bases from the raw reads, clean reads were obtained and aligned to the B73 maize genome (ZmB73_RefGen_v3) using BWA program with parameters set at mem -t 4 -k 32 –M [21, 22]. Next, duplications of clean reads were removed using SAMtools (v1.3) with parameter set at rmdup [23]. The polymorphic loci in populations were detected with Bayesian model, then the high quality SNPs were screened with miss rate <10%, minimum allele frequency (MAF) ≧0.05. SNPs location and its type detection was done using ANNOVAR software (Version 2013-05-20) [24]. Finally, the high quality SNPs were applied to genotype 80 maize lines used in the study.

### GWAS

A GWAS was performed using Fixed and random model Circulating Probability Unification [25] (FarmCPU). The threshold selection was related to the number of SNP markers.Extremely significant level was calculated by the following formula: 0.01/SNP_num. After converting, P<0.000001 was the significance threshold, at that level, the SNP markers were extremely significantly associated with the leaf angle and leaf orientation values. PLINK software [26] was used to calculate the average LD decay distance of the GWAS population.

### Candidate gene prediction

According to their physical locations, the SNP markers significantly associated with the leaf angle and leaf orientation values were compared with the B73 genome, and candidate genes for the two traits were detected within the LD range. The functions of candidate genes encoding production-related traits were annotated and predicted using MaizeGDB and NCBI. The unknown functions of candidate genes encoding production-related were annotated and predicted using the Arabidopsis database TAIRP43 [27], based on the similarity principle of homologous genes.

### Identified expression differences in candidate genes correlated with functional differences in phenotype

We verified 8 candidate genes for two traits based on the presence of significant SNPs within or less than 100 bp from the candidate genes and phenotype contribution rates greater than 8%. Some inbred maize lines from the GWAS population and biparental mapping population were selected as verification materials, according to significant differences in leaf angle and leaf orientation values. In the GWAS population, there were 8 inbred lines: W60, W10, W72, W54, W42, W34, W43 and W2 for verifying leaf angle and W60, W10, W72, W54, W42, W34, W43 and W16 for verifying leaf orientation values. In the biparental mapping population, 8 inbred lines (♀ P014, L130, L036, L008; ♂ E1312, L164, L108 and L047) were used to verify the leaf angle and (♀ P014, L130, L015, L017; ♂ E1312, L164, L144 and L047) the leaf orientation values. Total RNA was extracted from silking stage (R1) leaves in inbred lines. The expression of candidate genes was measured using quantitative real-time PCR [28] (qRT-PCR).

## Results and analysis

### Determination of phenotype data

#### Statistical analysis of the leaf angles and leaf orientation values of the GWAS population

**Fig 1:**
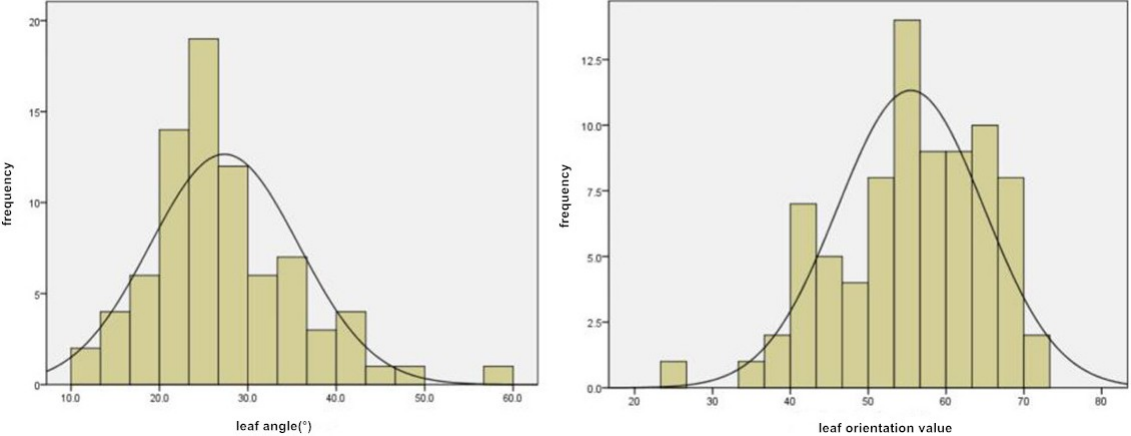
Distribution of leaf angle and leaf orientation value phenotypes in the GWAS population.

In 2015 and 2016, the mean leaf angle values in the GWAS population were 27.3° and 26.4°, respectively. The variation ranged from 12.7° to 55.7°. The coefficients of variation were 0.31 and 0.32, respectively. The mean leaf orientation values were 55.5 and 54.9, respectively, and the variation ranged from 23.4 to 72.9. The coefficients of variation were 0.17 and 0.22, respectively, and the phenotype data conformed to a normal distribution (Fig 1). The differences in leaf angles and leaf orientation values in this population were extremely significant (P<0.01).

#### Statistical analysis of the leaf angles and leaf orientation values of the biparental mapping population

**Fig 2:**
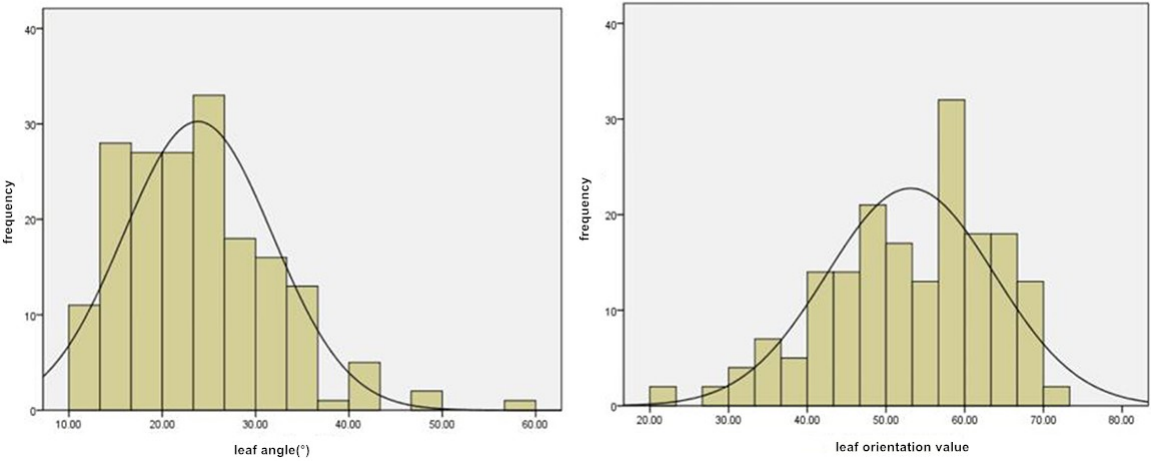
Distribution of leaf angle and leaf orientation value phenotypes in the biparental mapping population.

In 2015 and 2016, the mean leaf angle values in the biparental mapping population were 24.9° and 23.8°, respectively. The variation ranged from 10.9° to 54.9°. The coefficients of variation were 0.31 and 0.34, respectively. The mean leaf orientation values were 58.8 and 54.1, respectively, and the variation ranged from 29.7 to 72.8. The coefficients of variation were 0.14 and 0.21, respectively, and the phenotype data conformed to a normal distribution (Fig 2). The differences in leaf angles and leaf orientation values in this population were extremely significant (P<0.01).

### NGS quality analysis

#### NGS quality

A total of 3230.75 Gb of high-quality genomic data were obtained by NGS of the population, with an average of 40.38 Gb per sample, all the sequences have been deposited in the Sequence Read Archive (https://www.ncbi.nlm.nih.gov/sra) under the accession PRJNA495031. The average ratio of the population samples was 98.82%, the average sequencing depth of the genome was 17.62, and the average coverage was 88.39%. The data met the requirements for subsequent sequencing analysis in terms of data sequence depth and coverage.

#### Screening of SNPs and LD analysis

**Fig 3:**
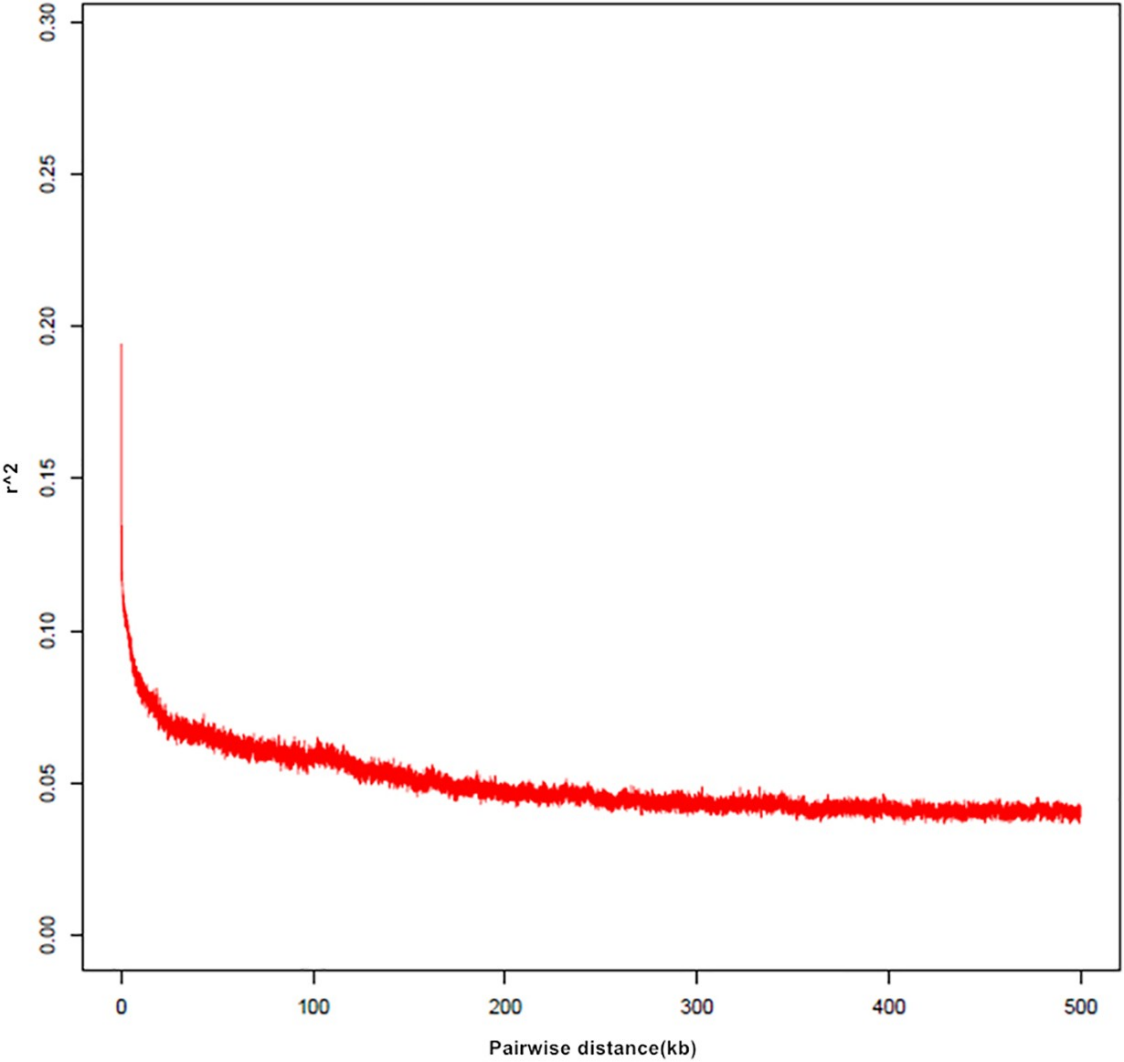
GWAS population LD decay map.

The 34872961 SNPs obtained from sequencing were filtered, with a miss rate of less than 10% and a MAF greater than 0.05. After screening and filtering, 1490007 SNPs were obtained for subsequent analysis. When the LD average decay distance (R^2^) was set to 0.1, the mean average decay distance in the GWAS population was 9.7 kb around the upper and lower SNPs, as determined after scanning the candidate genes within a 10-kb window (Fig 3).

### Genome-wide association analysis

#### Screening of SNPs significantly associated with leaf angle

**Fig 4:**
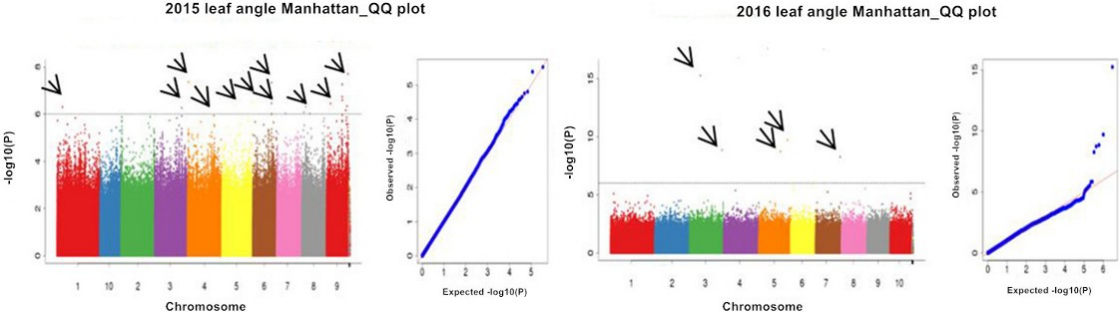
Leaf angle Manhattan plot and QQ plot for the two years.

Using P<0.000001 as a standard (Fig 4), from 2015 to 2016, a total of 22 SNP markers were significantly associated with the leaf angle (Table 1), and these markers were distributed on chromosomes 1, 3, 4, 5, 6, 7, 8, and 9. Among these markers, 17 significant SNPs were detected in 2015, with phenotype contribution rates ranging from 0.2% to 13.13%. Two SNPs, sLA-150000363 and sLA-143426315, were physically located within the candidate genes. sLA-150000363 was located in an exon of the candidate gene, conferring a synonymous mutation, and in 2015, a common significant correlation with leaf angle and leaf orientation values was observed. sLA-143426315 was located within an intron of the candidate gene. Five significant SNPs were detected in 2016, with phenotype contribution rates ranging from 4.55% to 21.62%. sLA-168123559 was located in an exon of the candidate gene, conferring a nonsynonymous mutation in the candidate gene *GRMZM2G039583*, which showed a glutamine (Q) to histidine (H) mutation at amino acid 369.

**Table 1:** Significant SNPS and candidate genes associated with leaf angle between 2015 and 2016.

Year	SNP	Physical position	Chr	Genotype	MAF	-log10(P)	Contribution (r^2^)	Candidate gene	Distance	Functional annotation	Reference
2015	sLA-38074392	38074392	1	C/T	0.1	6.3	4	GRMZM2G151521	8924		Chang et al. [29]
	sLA-190373170	190373170	3	C/T	0.32	6.27	9.12	GRMZM2G138800	-6954		Chang et al. [29]
			3	C/T	0.32	6.27		GRMZM2G440529	-46	Transcription factor bHLH112	
	sLA-5297715	5297715	4	G/A	0.11	7.37	3.85	GRMZM2G529003	8930		Kim et al. [30]
	sLA-86175016	86175016	5	A/T	0.05	6.37	1.9	GRMZM2G082683	-6121		Ku et al. [31]
	sLA-213570707	213570707	5	C/T	0.05	6.53	1				
	sLA-133124234	133124234	6	A/G	0.22	7.34	1.4	GRMZM2G155332	-9613		
	sLA-61074844	61074844	7	A/G	0.08	6.03	0.2				
	sLA-14706069	14706069	8	C/T	0.08	6.08	8.37	GRMZM2G519073	96	HSP20-like chaperone superfamily protein	Ku et al. [31]
	sLA-27862962	27862962	8	G/A	0.09	6.31	2.9				
	sLA-55427525	55427525	8	G/A	0.14	6.03	2.2				
	sLA-27812823	27812823	9	T/A	0.28	6.45	2.38				
	sLA-109965838	109965838	9	A/T	0.22	6.73	3.42				
	sLA-110028071	110028071	9	C/T	0.21	7.26	2.4				
	sLA-110879419	110879419	9	T/C	0.28	6.59	5.15	AC212747.3_FGT005	-751		
			9	T/C	0.28	6.59		GRMZM2G170148	9354		
			9	T/C	0.28	6.59		GRMZM2G472077	6585		
			9	T/C	0.28	6.59		GRMZM2G472087	1870		
	sLA-135003208	135003208	9	A/G	0.17	6.16	0.3	GRMZM2G024996	-313		Ku et al. [31]
	sLA-143426315	143426315	9	G/A	0.28	6.34	12.31	GRMZM2G147534	5301		
			9	G/A	0.28	6.34		GRMZM2G147671	0	26S proteasome non-ATPase regulatory subunit 4 homolog	Ku et al. [31]
			9	G/A	0.28	6.34		GRMZM2G147775	-5457		
	sLA-150000363	150000363	9	G/C	0.28	7.71	13.13	GRMZM2G400784	0	putative RING zinc finger domain superfamily protein	
2016	sLA-74714392	74714392	3	A/G	0.31	15.26	7.68				
	sLA-222474141	222474141	3	C/A	0.05	8.84	4.55				
	sLA-146719710	146719710	5	C/T	0.29	8.73	6.89	GRMZM2G411893	2448		Kim et al. [30]
	sLA-194375733	194375733	5	T/C	0.07	9.71	5.72				
	sLA-168123559	168123559	7	C/A	0.18	8.27	21.62	AC195147.3_FGT008	1163		Ku et al. [31]
			7	C/A	0.18	8.27		GRMZM2G039583	0	SNARE-associated Golgi protein family	
			7	C/A	0.18	8.27		GRMZM2G335738	-7338		
			7	C/A	0.18	8.27		GRMZM2G503990	4093		
			7	C/A	0.18	8.27		GRMZM6G522911	6231		

#### Screening of SNPs significantly associated with leaf orientation value

**Fig 5:**
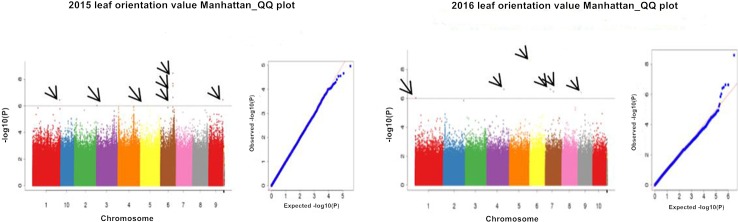
Leaf orientation value Manhattan plot and QQ plot for the two years.

Using P<0.000001 as a standard (Fig 5), between 2015 and 2016, a total of 11 SNP markers were significantly associated with the leaf orientation value (Table 2), and these SNPs were distributed on chromosomes 1, 3, 4, 5, 6, 7, and 9. Among these markers, 5 significant SNPs were detected in 2015, with phenotype contribution rates ranging from 3.79% to 20.15%. sLOV-150000363 was located in an exon of the candidate gene, conferring a synonymous mutation. Six significant SNPs were detected in 2016, with phenotype contribution rates ranging from 2.2% to 29.63%. Two SNPs, sLOV-6165883 and sLOV-182906583, were physically located within the candidate gene. sLOV-6165883 was located within an intron of the candidate gene. sLOV-182906583 was located in an exon of the candidate gene, conferring a nonsynonymous mutation in the candidate gene *GRMZM2G361659*, which showed an arginine (R) to threonine (T) mutation at amino acid 321.

**Table 2:** Significant SNPs and candidate genes associated with leaf orientation value between 2015 and 2016.

Year	SNP	Physical position	Chr	Genotype	MAF	-log10(P)	Contribution (r^2^)	Candidate gene	Distance	Functional annotation	Reference
2015	sLOV-133124234	133124234	6	A/G	0.22	8.46	6.79	GRMZM2G155332	-9613		
	sLOV-291544744	291544744	1	A/G	0.15	6.44	4.54				
	sLOV-4798376	4798376	3	T/A	0.18	6.03	3.79				
	sLOV-73732853	73732853	5	A/G	0.21	6.03	7.41				Ku et al. [31]
	sLOV-150000363	150000363	9	G/C	0.28	6.47	20.15	GRMZM2G400784	0	putative RING zinc finger domain superfamily protein	Ku [32]
2016	sLOV-53602402	53602402	7	A/G	0.06	6.64	5.12				Kim et al. [30]
	sLOV-86502393	86502393	7	G/A	0.23	6.48	6.15				
	sLOV-32513341	32513341	9	C/T	0.33	6.41	5.26				
	sLOV-6165883	6165883	1	G/C	0.43	6.04	29.63	GRMZM2G143588	0	DNA polymerase delta small subunit	Zhang [33]
	sLOV-182906583	182906583	4	G/C	0.36	6.64	27.49	GRMZM2G361659	0	E2F transcription factor-like E2FE	Ku et al. [31]
		182906583	4	G/C	0.36	6.64	2.2	GRMZM2G361693	-2426		
	sLOV-4904831	4904831	6	C/T	0.05	8.6	5.37	GRMZM2G391364	4533		
		4904831	6	C/T	0.05	8.6		GRMZM5G852185	7279		

### Candidate genes detected and analyzed

#### Leaf angle candidate gene detection and functional annotation

A total of 22 SNPs were scanned and analyzed between the two years, showing associations with leaf angle in 22 candidate genes (Table 1). *GRMZM2G039583* (SNP sLA-168123559 located within the candidate gene, with a phenotype contribution rate of 21.62%) encodes a SNARE protein associated with cell signaling pathways. *GRMZM2G147671* (SNP sLA-143426315 located within the candidate gene, with a phenotype contribution rate of 12.31%) encodes a 26S proteasome non-ATPase regulatory subunit. In 2015, screening for both the leaf angle and leaf orientation value showed that *GRMZM2G400784* (SNP sLA-150000363 located within the candidate gene, with a phenotype contribution rate of 13.13%) encodes a putative RING zinc finger domain superfamily protein. *GRMZM2G440529* (SNP sLA-190373170 located 46 bp from the candidate gene, with a phenotype contribution rate of 9.12%) encodes the transcription factor bHLH112. *GRMZM2G519073* (SNP sLA-14706069 located 96 bp from the candidate gene, with a phenotype contribution rate of 8.37%) encodes an HSP20-like chaperone superfamily protein.

#### Leaf orientation value candidate gene detection and functional annotation

Between the two years, 11 SNPs were scanned and analyzed to determine the associations with leaf orientation value, and 7 candidate genes were detected (Table 2). *GRMZM2G400784* (SNP sLOV-150000363 located within the candidate gene, with a phenotype contribution rate of 20.15%) encodes a putative RING zinc finger domain superfamily protein and was identified in screens for both leaf angle and leaf orientation value in 2015. *GRMZM2G143588* (SNP sLOV-6165883 located within the candidate gene, with a phenotype contribution rate of 29.63%) encodes a DNA polymerase delta small subunit. *GRMZM2G361659* (SNP sLOV-182906583 located within the candidate gene, with a phenotype contribution rate of 27.49%) encodes an E2F transcription factor.

### Partial verification of candidate genes

#### Identified expression differences in candidate genes correlated with functional differences in leaf angle phenotype

The expression levels of the 5 genes (*GRMZM2G039583*, *GRMZM2G147671*, *GRMZM2G400784*, *GRMZM2G440529* and *GRMZM2G519073*) were measured using qRT-PCR. The functions of the candidate genes were verified using comparative analysis.

#### Leaf angle candidate genes in the GWAS population were verified using qRT-PCR

In the GWAS population, 4 inbred maize lines with a mean leaf angle of less than 15.5° (W60, mean leaf angle of 12.75°; W10, mean leaf angle of 13.55°; W72, mean leaf angle of 15.25°; and W54 mean leaf angle of 15.25°) and another 4 inbred maize lines with a mean leaf angle of greater than 39° (W42, mean leaf angle of 55.75°; W34, mean leaf angle of 48.55°; W43, mean leaf angle of 43.5°; and W2, mean leaf angle of 39°) were selected to verify the candidate genes. Using qRT-PCR, the expression levels of *GRMZM2G039583*, *GRMZM2G147671*, *GRMZM2G400784*, *GRMZM2G440529* and *GRMZM2G519073* were determined.

**Fig 6:**
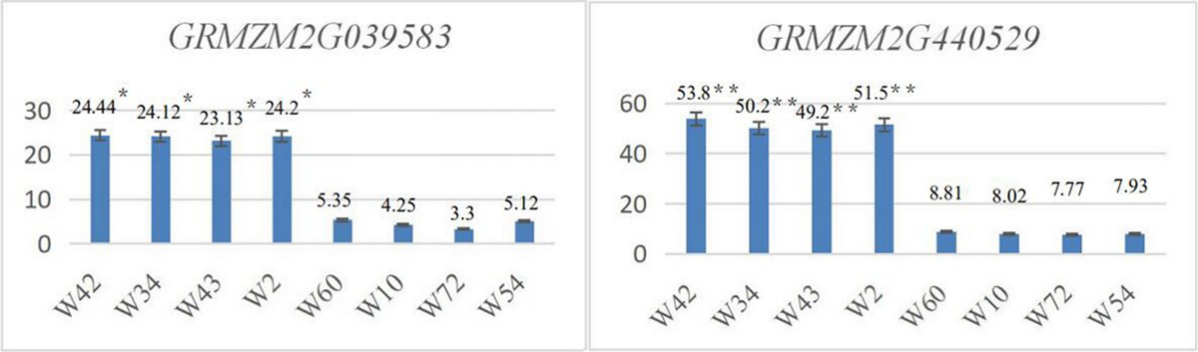
*GRMZM2G039583* and *GRMZM2G440529* qRT-PCR expression levels in the GWAS population.

**Fig 7:**
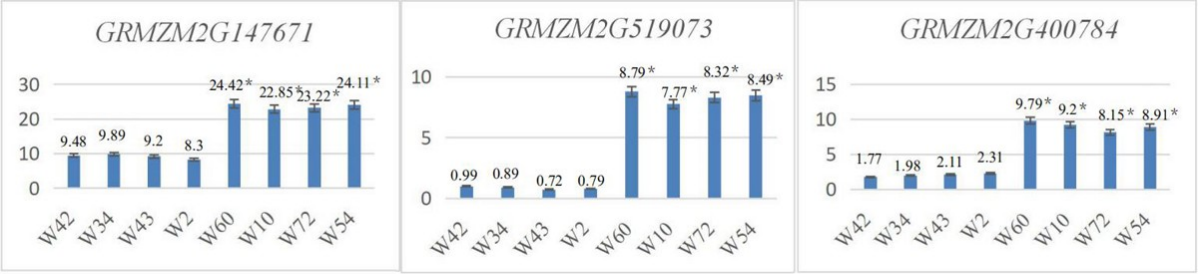
*GRMZM2G147671*, GRMZM2G519073 and *GRMZM2G400784* qRT-PCR expression levels in the GWAS population.

The results showed that the expression levels of the candidate genes *GRMZM2G039583* and *GRMZM2G440529* in inbred maize lines with larger leaf angles (W42, W34, W43, and W2) were significantly higher (P<0.05) than those of maize inbred lines with lower leaf angles (W60, W10, W72, and W54) (Fig 6). The expression levels of the candidate genes *GRMZM2G147671*, *GRMZM2G519073* and *GRMZM2G400784* in inbred maize lines with lower leaf angles (P<0.05) were significantly higher than those in inbred maize lines with larger leaf angles (Fig 7).

#### Leaf angle candidate genes in the biparental mapping population were verified using qRT-PCR

In the biparental mapping population, 4 inbred maize lines with mean leaf angles less than 23° (population ♀: P014, mean leaf angle of 22.9°; L130, mean leaf angle of 10.9°; L036, mean leaf angle of 11.7°; and L008, mean leaf angle of 11.5°) and another 4 inbred maize lines with mean leaf angles greater than 42° (population ♂: E1312, mean leaf angle of 54.9°; L164, mean leaf angle of 45.8°; L108, mean leaf angle of 42.5°; and L047, mean leaf angle of 42.3°) were selected to verify the candidate genes. Using qRT-PCR, the mean expression levels of *GRMZM2G039583*, *GRMZM2G147671*, *GRMZM2G440529*, *GRMZM2G519073* and *GRMZM2G400784* were determined.

**Fig 8:**
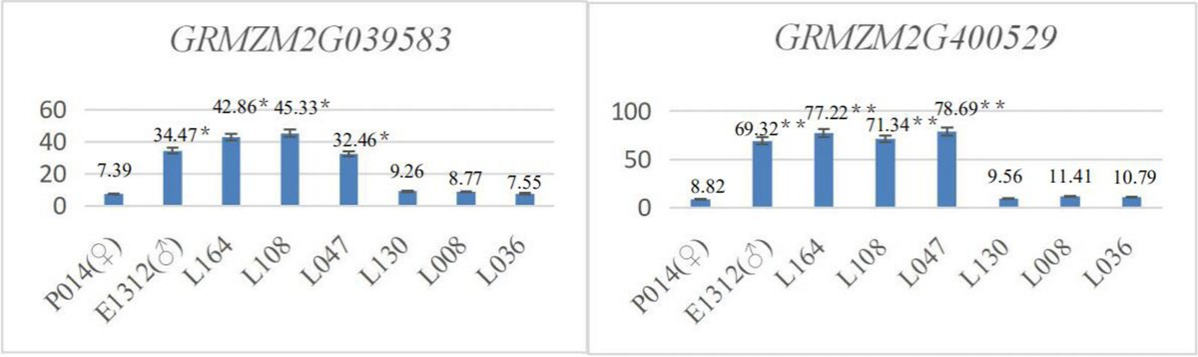
*GRMZM2G039583* and *GRMZM2G440529* qRT-PCR expression levels in the biparental mapping population.

**Fig 9:**
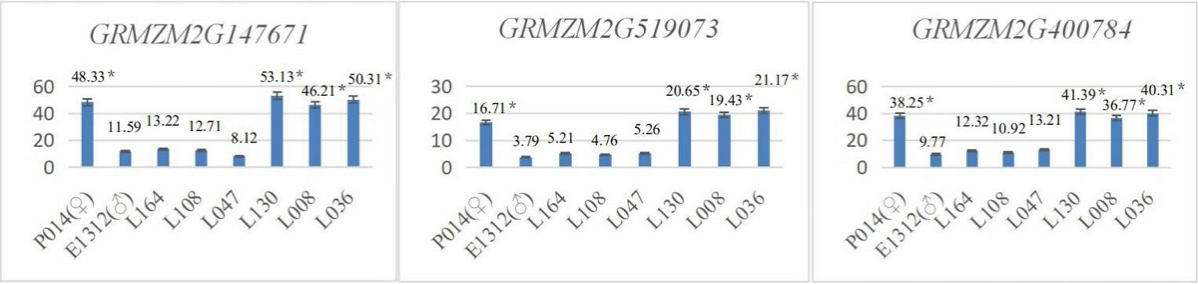
*GRMZM2G147671*, *GRMZM2G519073* and *GRMZM2G400784* qRT-PCR expression levels in the biparental mapping population.

The results showed that the expression levels of the candidate genes *GRMZM2G039583* and *GRMZM2G440529* in inbred maize lines with larger leaf angles (♂ E1312, L164, L108, and L047) were significantly higher (P<0.05) than those in maize inbred lines with lower leaf angles (♀ P014, L130, L036, and L008) (Fig 8). The expression levels of the candidate genes *GRMZM2G147671*, *GRMZM2G519073* and *GRMZM2G400784* in maize inbred lines with lower leaf angles were significantly higher (P<0.05) than those in maize inbred lines with larger leaf angles (Fig 9).

#### Identified expression differences in candidate genes that correlated with functional differences in the leaf orientation value phenotype

The expression levels of 3 genes, *GRMZM2G400784*, *GRMZM2G143588* and *GRMZM2G361659*, were measured using qRT-PCR. The functions of the candidate genes were verified using comparative analysis.

#### Leaf orientation value candidate genes in the GWAS population were verified using qRT-PCR

In the GWAS population, 4 inbred maize lines with mean leaf orientation values greater than 69 (W60, mean leaf orientation value of 72.9; W10, mean leaf orientation value of 69.7; W72, mean leaf orientation value of 70.6; and W54, mean leaf orientation value of 70.8) and another 4 inbred maize lines with mean leaf orientation values less than 33 (W42, mean leaf orientation value of 23.4; W34, mean leaf orientation value of 30.1; W43, mean leaf orientation value of 32.3; and W16, mean leaf orientation value of 31.8) were selected to verify the candidate genes. Using qRT-PCR, the expression levels of *GRMZM2G143588*, *GRMZM2G361659* and *GRMZM2G400784* were determined.

**Fig 10:**
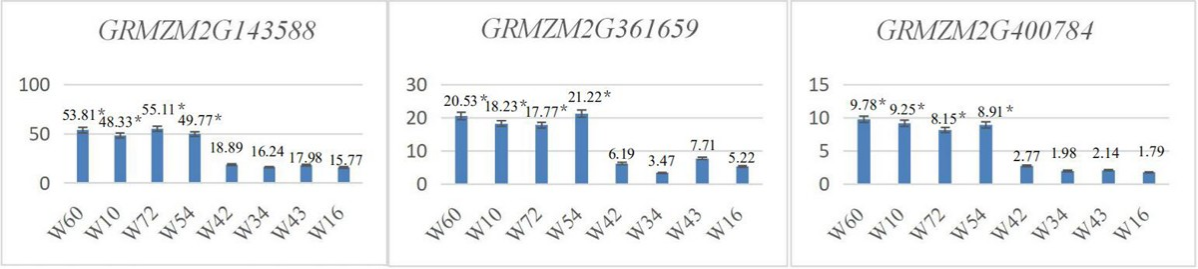
*GRMZM2G143588*, *GRMZM2G361659* and *GRMZM2G400784* qRT-PCR expression levels in the GWAS population.

The results showed that the expression levels of the candidate genes *GRMZM2G143588*, *GRMZM2G361659* and *GRMZM2G400784* in maize inbred lines with higher leaf orientation values (W60, W10, W72, and W54) were significantly h**i**gher (P<0.05) than those in inbred maize lines with lower leaf orientation values (W42, W34, W43, and W16) (Fig 10).

#### Leaf orientation value candidate genes in the biparental mapping population were verified using qRT-PCR

In the biparental mapping population, 4 inbred maize lines with mean leaf orientation values greater than 64 (♀: P014, mean leaf orientation value of 64.6; L130, mean leaf orientation value of 71.8; L017, mean leaf orientation value of 70.7; and L015, mean leaf orientation value of 72.8) and another 4 inbred maize lines with mean leaf orientation values less than 33 (♂: E1312, mean leaf orientation value of 32.3; L164, mean leaf orientation value of 29.7; L144, mean leaf orientation value of 29.9; and L047, mean leaf orientation value of 31.8). Using qRT-PCR, the expression levels of *GRMZM2G143588*, *GRMZM2G361659* and *GRMZM2G400784* were determined.

**Fig 11:**
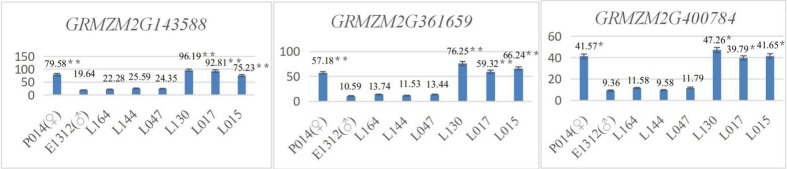
*GRMZM2G143588*, *GRMZM2G361659* and *GRMZM2G400784* qRT-PCR expression levels in the biparental mapping population.

The results showed that the expression levels of the candidate genes *GRMZM2G143588*, *GRMZM2G361659* and *GRMZM2G400784* in inbred maize lines with higher leaf orientation values (♀ P014, L130, L017, and L015) were significantly higher (P<0.05) than those of maize inbred lines with lower leaf orientation values (♂ E1312, L164, L144, and L047) (Fig 11).

## Discussion

### SNP markers for leaf angle and leaf orientation

In the present study, GWAS analysis was used to directly locate SNPs significantly associated with leaf angle and leaf orientation value, thereby improving location accuracy. The confidence intervals of the published QTLs associated with plant type were high (approximately 10^6^−10^8^ bp [31]). In the GWAS population, the average decay distance was 9.7 kb, which greatly improved the scanning accuracy of the candidate genes. SSR markers localized the leaf traits with only approximately 20 primer pairs per chromosome [34]. In the present study, 1.8×10^5^ SNPs were equally distributed among the chromosomes for the GWAS; chromosome 1 harbored approximately 3.0×10^5^ SNPs, while chromosome 10 harbored approximately 1.2×10^5^ SNPs.

In the present study, 5 SNPs were significantly associated with the leaf angle, and the phenotype contribution rate was higher than 8%. Three of these genes were consistent with published QTLs. sLA-190373170 was 1.9 Mb away from an important region, which controlled the leaf angle gene umc1311-bnlg1160 identified by Chang et al. [29]. sLA-14706069 was 2.1 Mb from an important region, consistent with Ku et al. [31], who identified an important region controlling the leaf angle gene umc1304-phi121. sLA-143426315 was consistent with Ku et al. [31], who identified an important region controlling the leaf angle gene bnlg1525-umc2095. All 3 SNPs were significantly associated with the leaf orientation value, and the phenotype contribution rate was higher than 8%, which was consistent with published QTLs. sLOV-150000363, which was significantly correlated with leaf angle and leaf orientation value, was 0.12 Mb; this finding was consistent with Ku [32], who identified an important region controlling the leaf orientation value; in addition, bnlg619-IDP8193.sLOV-6165883 was 0.8 Mb, which was consistent with Liu et al. [34], who identified region bnlg1014.sLOV-182906583, which was 2.5 Mb, consistent with Ku et al. [31], who identified region umc1086-umc2289. In addition, unreported SNP markers that were significantly associated with leaf angle and leaf orientation values were detected, probably due to the multigene inheritance of corn leaf angle and leaf orientation values. The parental QTL mapping used by previous investigators made it difficult to locate microgenes.

In the present study, we screened 2 significant SNPs in the exons of leaf angle candidate genes: sLA-150000363 conferred a synonymous mutation, and sLA-168123559 conferred a nonsynonymous mutation. Two significant SNPs were detected in the exons of leaf orientation value candidate genes: sLOV-150000363 conferred a synonymous mutation, and sLOV-182906583 conferred a nonsynonymous mutation. These newly screened significant SNPs located in the exons of candidate genes provide valuable data for further investigating the molecular genetic basis of leaf angle and leaf orientation value.

### Functional analysis of partial candidate genes

Among SNPs that were inside or less than 100 bp from candidate genes and had phenotype contribution rates greater than 8%, we screened 8 candidate genes associated with leaf angle and leaf orientation value. Using the TAIRP43 database to predict the functions of the candidate genes, we observed that *GRMZM2G400784* regulated both leaf angle and leaf orientation value, acting as a negative regulator in the GWAS population and biparental mapping population inbred lines with lower leaf angles, and the expression level was significantly higher than that in populations with higher leaf angles. The leaf orientation value acted as a positive regulator in the GWAS population and biparental mapping population inbred lines with higher leaf orientation values, and the expression level was significantly higher than that in populations with lower leaf orientation values. Functional annotation revealed a putative RING zinc finger domain superfamily protein that may play an important role in gene expression regulation and cell differentiation [30]. The leaf angle gene *GRMZM2G039583*, which functions in positive regulation, encodes a SNARE protein that is associated with cellular signaling pathways; this protein promotes the formation of plant cell plates and interacts with ion channel proteins that regulate the normal growth and development of plants by forming a complex [35]. The leaf angle gene *GRMZM2G147671*, which acts in negative regulation, encodes a 26S proteasome non-ATPase regulatory subunit; the 26S proteasome pathway is important in the polarity of the adaxial surface of leaf, and polarity formation may be associated with leaf morphogenesis [36]. The leaf angle gene *GRMZM2G440529*, which functions in positive regulation, belongs to the BHLH112 transcription factor family, which may regulate growth and development, nutrient absorption, signal transduction, and resistance to stress through homologous or heterologous diploid forms [37]. *GRMZM2G519073*, which functions as a negative regulator of leaf angle, was annotated as an HSP20 heat shock protein, a major chaperone that regulates cell growth and development by directing the proper assembly of other proteins [38].

The leaf orientation value gene *GRMZM2G143588*, which acts as a positive regulator, encodes a DNA polymerase delta small subunit. *GRMZM2G361659*, a positive regulator of leaf orientation value, belongs to the E2F transcription factor family, and E2Fe transcription factors may regulate the replication of DNA by regulating the ploidy of the chromosomes during plant cell proliferation [39].

## Conclusion

The present study utilized a GWAS population containing 1.49×10^6^ single SNP markers, combining phenotype data from two years in a GWAS, and identified a total of 33 SNPs significantly associated (P<0.000001) with two target traits. Among these markers, 1 SNP was significantly associated with both traits. A total of 22 SNPs were significantly associated with the leaf angle, and 5 SNPs showed phenotype contribution rates greater than 8%. A total of 11 SNPs were significantly associated with leaf orientation value, and 3 SNPs showed phenotype contribution rates greater than 8%.

Screening revealed two significant SNPs in the exons of leaf angle candidate genes, sLA-150000363 and sLA-168123559, and sLA-143426315 was located in an intron of the candidate gene. Screening revealed two significant SNPs in the exons of leaf orientation value candidate genes, sLOV-150000363 and sLOV-18290658, and sLOV-6165883 was located in an intron of the candidate gene.

In the LD region of the significant SNP locus, 22 leaf angle candidate genes were detected, and among SNPs inside or within 100 bp of the candidate gene that had a phenotype contribution rate greater than 8%, the expression levels of five candidate genes *GRMZM2G039583*, *GRMZM2G147671*, *GRMZM2G400784*, *GRMZM2G440529* and *GRMZM2G519073* were detected in the GWAS population and biparental mapping population. Two candidate genes, *GRMZM2G039583* and *GRMZM2G440529*, act as positive regulators of leaf angle, and *GRMZM2G039583* encodes a SNARE family protein that is associated with cellular signaling pathways that regulate the normal growth and development of plants. *GRMZM2G440529* encodes a BHLH112 transcription factor, which may regulate growth and development, nutrient absorption and signal transduction through homologous or heterologous diploid forms. Three candidate genes, *GRMZM2G147671*, *GRMZM2G519073* and *GRMZM2G400784*, functioned as negative regulators of leaf angle. *GRMZM2G147671* encodes a 26S proteasome non-ATPase regulatory subunit, which promotes the polarity of the adaxial surface of the leaf, and polarity formation may be associated with leaf morphogenesis. *GRMZM2G519073* encodes an HSP20 heat shock protein that regulates cell growth and development by directing the proper assembly of proteins. *GRMZM2G400784* encodes a putative RING zinc finger domain superfamily protein that may play an important role in gene expression regulation and cell differentiation.

In the LD region of the significant SNP locus, 7 leaf orientation value candidate genes were detected, and the expression levels of three candidate genes, *GRMZM2G400784*, *GRMZM2G143588* and *GRMZM2G361659*, were detected in the GWAS population and biparental mapping population. The three candidate genes act as positive regulators of leaf orientation value. Thus, *GRMZM2G400784* regulated both leaf angle and leaf orientation value, acting as a negative regulator of leaf angle and a positive regulator of leaf orientation value. *GRMZM2G143588* encodes a DNA polymerase delta small subunit. *GRMZM2G361659* encodes a member of the E2F transcription factor family, which may regulate the replication of DNA by regulating the ploidy of the chromosomes during plant cell proliferation.

These results lay the foundation for analyzing the genetic mechanisms of plant types in spring maize, enriching our understanding of the molecular genetic mechanism of maize plant types.
